# Validation of *Cis* and *Trans* Modes in Multistep Phosphotransfer Signaling of Bacterial Tripartite Sensor Kinases by Using Phos-Tag SDS-PAGE

**DOI:** 10.1371/journal.pone.0148294

**Published:** 2016-02-01

**Authors:** Emiko Kinoshita-Kikuta, Eiji Kinoshita, Yoko Eguchi, Tohru Koike

**Affiliations:** 1 Department of Functional Molecular Science, Institute of Biomedical and Health Sciences, Hiroshima University, Hiroshima, Japan; 2 Department of Science and Technology on Food Safety, Faculty of Biology-Oriented Science and Technology, Kinki University, Kinokawa, Japan; University of Padova, ITALY

## Abstract

Tripartite sensor kinases (TSKs) have three phosphorylation sites on His, Asp, and His residues, which are conserved in a histidine kinase (HK) domain, a receiver domain, and a histidine-containing phosphotransmitter (HPt) domain, respectively. By means of a three-step phosphorelay, TSKs convey a phosphoryl group from the γ-phosphate group of ATP to the first His residue in the HK domain, then to the Asp residue in the receiver domain, and finally to the second His residue in the HPt domain. Although TSKs generally form homodimers, it was unknown whether the mode of phosphorylation in each step was intramolecular (*cis*) or intermolecular (*trans*). To examine this mode, we performed *in vitro* complementation analyses using Ala-substituted mutants of the ATP-binding region and three phosphorylation sites of recombinant ArcB, EvgS, and BarA TSKs derived from *Escherichia coli*. Phosphorylation profiles of these kinases, determined by using Phos-tag SDS-PAGE, showed that the sequential modes of the three-step phosphoryl-transfer reactions of ArcB, EvgS, and BarA are all different: *cis*-*trans*-*trans*, *cis*-*cis*-*cis*, and *trans*-*trans*-*trans*, respectively. The inclusion of a *trans* mode is consistent with the need to form a homodimer; the fact that all the steps for EvgS have *cis* modes is particularly interesting. Phos-tag SDS-PAGE therefore provides a simple method for identifying the unique and specific phosphotransfer mode for a given kinase, without taking complicated intracellular elements into consideration.

## Introduction

Bacterial cells possess phosphotransfer signaling mechanisms known as ‘two-component regulatory systems’ that elicit a variety of adaptive responses to the cells’ environments [1, 2]. Each of these systems generally consists of a histidine sensor kinase and a response regulator. The sensor kinase senses extra- and intracellular stimuli and regulates the function of its cognate response regulator through a phosphorylation reaction. Accordingly, the response regulator mediates certain changes in gene expression or cell behavior. Many instances of phosphotransfer signaling have been discovered, not only in bacterial prokaryotes, but also in diverse eukaryotic species, including yeasts, fungi, and plants [1]. Two-component-like signaling systems have not been discovered in mammals, although certain protein kinases for His residues and His-mediated phosphotransfer systems have been reported [3, 4]. A typical sensor kinase has a histidine kinase (HK) domain containing an invariant His residue that is autophosphorylated in an ATP-dependent manner, whereas a typical response regulator has a receiver domain containing a conserved Asp residue that can acquire a phosphoryl group from its cognate sensor kinase. Most two-component systems have this type of a simple His–Asp phosphorelay scheme. However, some histidine sensor kinases, known as tripartite sensor kinases (TSKs), have a more complex type of phosphorelay consisting of two additional domains: a receiver domain containing a conserved Asp residue, and a histidine-containing phosphotransmitter (HPt) domain. In such systems, signals are transmitted through a more sophisticated three-step phosphorelay. First, a phosphoryl group moves from ATP to the HK domain (His residue); secondly, it moves to the receiver domain (Asp residue); and finally it moves to the HPt domain (His residue). Subsequently, the HPt-phosphorylated TSKs phosphorylate the receiver domain (Asp residue) of response regulators.

**Fig 1:**
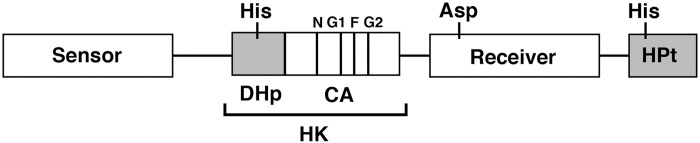
A schematic diagram of a monomer subunit of TSK showing a sensor domain, an HK domain, a receiver domain, and an HPt domain. The HK domain contains two subdomains: a DHp subdomain and a CA subdomain. The N, G1, F, and G2 boxes are conserved sequence motifs in histidine sensor kinases.

A schematic diagram of a monomer subunit of TSK is presented in Fig 1 [5, 6]. The autophosphorylation reaction of the sensor kinases usually takes place in homodimers. The HK domain is highly conserved and consists of two subdomains: a dimerization-inducing His-containing phosphotransmitter (DHp) subdomain and a catalytic and ATP-binding (CA) subdomain. The ATP-binding region of the CA subdomain contains four unique signature sequences known as the N, G1, F, and G2 boxes. The invariant His residue in the DHp subdomain is a primary autophosphorylation site. An early biochemical study using EnvZ, a typical and simple sensor kinase derived from *Escherichia coli*, demonstrated that the CA subdomain catalyzes transfer of the γ-phosphate group of ATP, which is bound to one monomer subunit, to the His residue in the DHp subdomain of the other subunit in the homodimer; this confirmed that the autophosphorylation reaction of EnvZ occurs in an intermolecular (*trans*) mode [7]. In the wake of this early study, it had been suggested that dimeric sensor kinases autophosphorylate by means of a *trans* mode [2]. Recently, however, the sensor kinases HK853 from *Thermotoga maritime*, PhoR from *Staphylococcus aureus*, and ArcB from *E*. *coli* have been shown to be autophosphorylated in a *cis* mode [8, 9]. Furthermore, it has been reported that a small loop within the DHp subdomain determines whether sensor kinases are autophosphorylated in a *cis* or a *trans* mode [10]; however, the mode of autophosphorylation in most sensor kinases has not been actually verified.

The mode of the three-step phosphorelay from ATP to the terminal HPt domain in TSKs remains unclear in most cases, although several reports have appeared in the literature. Some complementation assays using site-directed mutagenesis of phosphorylation sites have been performed to determine the phosphotransfer mode in TSKs of TorS from *E*. *coli* [11] and BvgS from *Bordetella pertussis* [12]. These assays showed that the TorS phosphotransfer reactions from the first His residue in the HK domain to the Asp residue in the receiver domain, and then to the second His residue in the HPt domain proceed through *trans* modes, whereas BvgS transfers a phosphoryl group from the first His residue to the Asp residue in a *cis* mode, and then from the receiver domain to the HPt domain in a *trans* mode. Regarding the phosphotransfer mode of BvgS, Cotter and Jones suggest that a phosphoryl group is transferred from the γ-phosphate of ATP to the HK domain in a *trans* mode, and that a *cis* mode is adopted throughout the subsequent steps of His–Asp–His phosphorelay [13]. These reports suggest that each individual TSK has a unique phosphorylation mode, and that a focused and reliable study is necessary to prove that a specific mode applies to a particular kinase.

In this study, we introduce Phos-tag SDS-PAGE as a simple method for identifying whether the reactions of autophosphorylation and phosphoryl transfer in TSKs occur in a *cis* or in a *trans* mode. Phos-tag SDS-PAGE is a technique for phosphate-affinity electrophoresis that is capable of separating multiple phosphoprotein species that contain identical numbers of phosphoryl groups, but in which the phosphoryl groups are attached at different locations within the protein molecules [14–19]. This method offers the following major advantages: (i) the phosphate-affinity procedure is almost identical to that for conventional SDS-PAGE; (ii) a downstream procedure, such as gel staining, Western blotting, or mass spectrometric analysis, can be applied; (iii) radioactive and chemical labels are unnecessary for kinase and phosphatase assays; (iv) various phosphoprotein species with differing phosphorylation statuses can be detected separately as multiple migration bands; (v) the phosphate-binding specificity is independent of the kind of phosphorylated amino acid; (vi) several phosphoprotein species having the same number of phosphate groups can be separated; (vii) the time-course of the quantitative ratio of phosphorylated to nonphosphorylated proteins can be determined; (viii) unstable His- and Asp-phosphorylated proteins involved in a two-component signaling system can be detected simultaneously during their phosphotransfer reactions; and (ix) three types of phosphorylated species in TSKs derived from the HK domain, the receiver domain, and the HPt domain, respectively, can be detected separately as three migration bands.

By using our original Phos-tag SDS-PAGE method, we performed *in vitro* complementation assays for ArcB, EvgS, and BarA TSKs from *E*. *coli* to establish the specific phosphorylation mode for each kinase. As a result, Phos-tag SDS-PAGE permitted us to identify a unique and specific phosphotransfer mode for a given kinase. We also discuss a variety of modes found in multistep phosphotransfer signaling mechanisms of TSKs.

## Materials and Methods

### Materials

The acrylamide-pendent Phos-tag ligand (AAL-107) is commercially available from Wako Pure Chemical Industries (Osaka, Japan). ATP and lithium potassium acetyl phosphate (AP) were purchased from Sigma-Aldrich (St. Louis, MO).

### Preparation of recombinant proteins derived from *E*. *coli*

To construct plasmids for overexpression of the cytoplasmic region of the sensor kinases EnvZ, ArcB, EvgS, and BarA and the corresponding mutants with Ala substitution in each G2 box or phosphorylation site, as well as full-length ArcA, EvgA, and UvrY, the corresponding DNA fragments were prepared by PCR using genome DNA of *E*. *coli* W3110 as a template in conjunction with a set of primer pairs. The sequences of the primers used in this study are listed in Table 1. After digestion of the PCR-amplified fragments with two types of restriction enzyme, each introducing a single cleavage within one of the primer pairs, the fragments were inserted into a pET21a(+)vector (Merck; Darmstadt, Germany) between the same restriction sites as those used for the preparation of the insert DNAs. Mutations were introduced into the G2 box or the phosphorylation sites of the sensor kinases by using a Quick-Change Site-Directed Mutagenesis Kit (Stratagene; La Jolla, CA). The sequence was confirmed by using an ABI PRISM 310 Genetic Analyzer (Applied Biosystems; Foster City, CA). Each constructed plasmid was transformed into *E*. *coli* BL21(DE3) or BL21(DE3)pLysS. The host cells were grown in Luria–Bertani broth at 37°C, and the targeted proteins were overexpressed by induction with isopropyl β-d-1-thiogalactopyranoside. N-Terminal proteins tagged on histidine residues were purified by using nickel–NTA agarose (Qiagen; Hilden, Germany). The purified proteins were stored in 10 mM Tris–HCl (pH 8.0) containing 50% (v/v) glycerol at –20°C.

**Table 1:** PCR primers used in this study.

Primer	Sequence (5'–3')	Function
EnvZ (truncated)_F	GTTTATTGCTGGATCCAACCGACCGTTGGT	Amplification of truncated EnvZ
EnvZ (truncated)_R	CTTCGCCTCCCGGCGGCCGCCCCTTGTTTT	Amplification of truncated EnvZ
EnvZ (G2*)_F	GCGCGCACCATTAGCGCCACGGCATTAGGGCTGGCAATTGTGCAG	Mutagenesis
EnvZ (G2*)_R	CTGCACAATTGCCAGCCCTAATGCCGTGGCGCTAATGGTGCGCGC	Mutagenesis
EnvZ (H243A)_F	ATGGCGGGGGTAAGTGCCGACTTGCGCACGCCG	Mutagenesis
EnvZ (H243A)_R	CGGCGTGCGCAAGTCGGCACTTACCCCCGCCAT	Mutagenesis
ArcB (truncated)_F	TTCTATCGGTGGTCGGATCCCAACTGGAGG	Amplification of truncated ArcB
ArcB (truncated)_R	GGTCTAGCGCGGCCGCTTTTTTAGTGGCTT	Amplification of truncated ArcB
ArcB (G2*)_F	CTGCCACCGCCACCGCTATTGCTCTGGCCG	Mutagenesis
ArcB (G2*)_R	CGGCCAGAGCAATAGCGGTGGCGGTGGCAG	Mutagenesis
ArcB (H292A)_F	ATCTCCACCATCAGTGCCGAATTGCGTACACCG	Mutagenesis
ArcB (H292A)_R	CGGTGTACGCAATTCGGCACTGATGGTGGAGAT	Mutagenesis
ArcB (D576A)_F	GACCTGGTGTTGCTGGCTATTCAGTTGCCAGAT	Mutagenesis
ArcB (D576A)_R	ATCTGGCAACTGAATAGCCAGCAACACCAGGTC	Mutagenesis
ArcB (H717A)_F	ATTGTTGAGGAAGGAGCTAAAATTAAAGGTGCG	Mutagenesis
ArcB (H717A)_R	CGCACCTTTAATTTTAGCTCCTTCCTCAACAAT	Mutagenesis
ArcA_F	CAATTTAGGTAGGATCCATGCAGACCCCGC	Amplification of ArcA
ArcA_R	ACGGTGGTAAAGCGGCCGCATCTTCCAGAT	Amplification of ArcA
EvgS (truncated)_F	TGGGGATTCTACGGATCCCGCTCAGTTCGT	Amplification of truncated EvgS
EvgS (truncated)_R	ATTGTGGGAGCCGCGGCCGCGTCATTTTTC	Amplification of truncated EvgS
EvgS (G2*)_F	AGCAAACAGCTTCTGCTTTAGCCTTAATGA	Mutagenesis
EvgS (G2*)_R	TGATTAAGGCTAAAGCAGAAGCTGTTTGCT	Mutagenesis
EvgS (H721A)_ F	TCTGGCAACGATGAGTGCCGAAAATAAGAACACCA	Mutagenesis
EvgS (H721A)_R	TGGTGTTCTTATTTTCGGCACTCATCGTTGCC	Mutagenesis
EvgS (D1009A)_F	GATCTGCTTATTACTGCCGTTAATATGCCGAA	Mutagenesis
EvgS (D1009A)_R	TTCGGCATATTAACGGCAGTAATAAGCAGATC	Mutagenesis
EvgS (H1137A)_F	TTCCATCAGTGTATTGCCCGCATCCACGGTGC	Mutagenesis
EvgS (H1137A)_R	GCACCGTGGATGCGGGCAATACACTGATGGAA	Mutagenesis
EvgA_F	CAAAGGGAAGGATCCATGAACGCAATAATT	Amplification of EvgA
EvgA_R	AAAAACTTCAGCGGCCGCGCCGATTTTGTT	Amplification of EvgA
BarA (truncated)_F	TTCTATCGGTGGTCGGATCCCAACTGGAGG	Amplification of truncated BarA
BarA (truncated)_R	GGTCTAGCGCGGCCGCTTTTTTAGTGGCTT	Amplification of truncated BarA
BarA (G2*)_F	GTCATGGTGCCACCGCTCTGGCGCTGGTGA	Mutagenesis
BarA (G2*)_R	CGGCCAGAGCAATAGCGGTGGCGGTGGCAG	Mutagenesis
BarA (H302A)_F	CTGGCAAATATGTCAGCCGAGCTGCGTACACCA	Mutagenesis
BarA (H302A)_R	TGGTGTACGCAGCTCGGCTGACATATTTGCCAG	Mutagenesis
BarA (D718A)_F	GATTTGATCTTAATGGCTATTCAAATGCCTGAC	Mutagenesis
BarA (D718A)_R	GTCAGGCATTTGAATAGCCATTAAGATCAAATC	Mutagenesis
BarA (H861A)_F	CTGGTTGATTTGATTGCTAAACTGCATGGCAGT	Mutagenesis
BarA (H861A)_R	ACTGCCATGCAGTTTAGCAATCAAATCAACCAG	Mutagenesis
UvrY_F	ATTTCTGGAGATGGATCCTTGATCAACGTT	Amplification of UvrY
UvrY_R	CGTCAAACTGGCGGCCGCCTGACTTGATAA	Amplification of UvrY

### *In vitro* phosphorylation assay

Autophosphorylation of EnvZ (0.4 mg/mL, 12.5 μM) was performed in 50 mM Tris–HCl (pH 8.0) containing 25 mM KCl, 5.0 mM MgCl_2_, and 10 mM ATP at 25°C for 10 min. The autophosphorylation reactions of ArcB and BarA, and the phosphorelay reactions of ArcB/ArcA and BarA/UvrY (each at 0.4 mg/mL; 4.8 μM ArcB and 15.0 μM ArcA, 4.9 μM BarA and 13.3μM UvrY) were performed in 50 mM Tris–HCl (pH 8.0) containing 25 mM KCl, 5.0 mM MgCl_2_, 10 mM DTT, and 10 mM ATP at 25°C for 10 min. Autophosphorylation of EvgS and the phosphorelay reaction of EvgS/EvgA (each at 0.4 mg/mL; 5.4 μM EvgS and 15.8 μM EvgA) were performed in 0.30 M Tris–HCl (pH 8.0) containing 50 mM KCl, 10 mM MgCl_2_, and 30 mM ATP at 25°C for 10 min. The AP-dependent autophosphorylation reactions of ArcB, EvgS, and BarA mutant proteins (each at 0.4 mg/mL; 4.8 μM ArcB, 5.4 μM EvgS, and 4.9 μM BarA) were performed in 0.30 M Tris–HCl (pH 8.0) containing 50 mM KCl, 10 mM MgCl_2_, and 40 mM AP at 25°C for 10 min. All reactions were carried out for 10 min, then terminated by adding a half volume of 3× sample-loading buffer for SDS-PAGE, consisting of 195 mM Tris–HCl (pH 6.8), 3.0% (w/v) SDS, 30% (v/v) glycerol, 15% (v/v) 2-sulfanylethanol, and 0.10% (w/v) bromophenol blue (BPB). Sample solutions were not boiled before electrophoresis.

### Phos-tag SDS-PAGE and gel staining

Electrophoresis was usually performed at 30 mA/gel and room temperature by using a 1-mm-thick, 9-cm-wide, and 9-cm-long gel on an AE-6500 PAGE apparatus (Atto; Tokyo, Japan). The separating gel (6.3 mL) consisted of 8% (w/v) polyacrylamide and 375 mM Tris–HCl buffer (pH 8.8), and the stacking gel (1.8 mL) consisted of 4% (w/v) polyacrylamide and 125 mM Tris–HCl buffer (pH 6.8). The acrylamide-pendent Phos-tag ligand (20 μM) and two equivalents of MnCl_2_ (40 μM) were added to the separating gel before polymerization. An acrylamide stock solution was prepared containing a 29:1 mixture of acrylamide and *N*,*N*'-methylenebisacrylamide. The running buffer consisted of 192 mM glycine and 25 mM Tris containing 0.10% (w/v) SDS. The electrophoresis was continued until the BPB dye reached the bottom of the separating gel. After electrophoresis, the gels were stained with a solution of colloidal CBB G-250. The CBB solution was prepared as follows. (i) Al_2_(SO_4_)_3_·14–18H_2_O (50 g) was dissolved in distilled water (800 mL); (ii) EtOH (100 mL) was added with stirring; (iii) CBB G-250 (0.20 g) was dissolved with stirring; (iv) 85% (v/v) phosphoric acid (24 mL) was added with stirring; and finally, (v) the solution was diluted to 1 L with distilled water.

## Results

### Intermolecular autophosphorylation of EnvZ verified by Phos-tag SDS-PAGE

**Fig 2:**
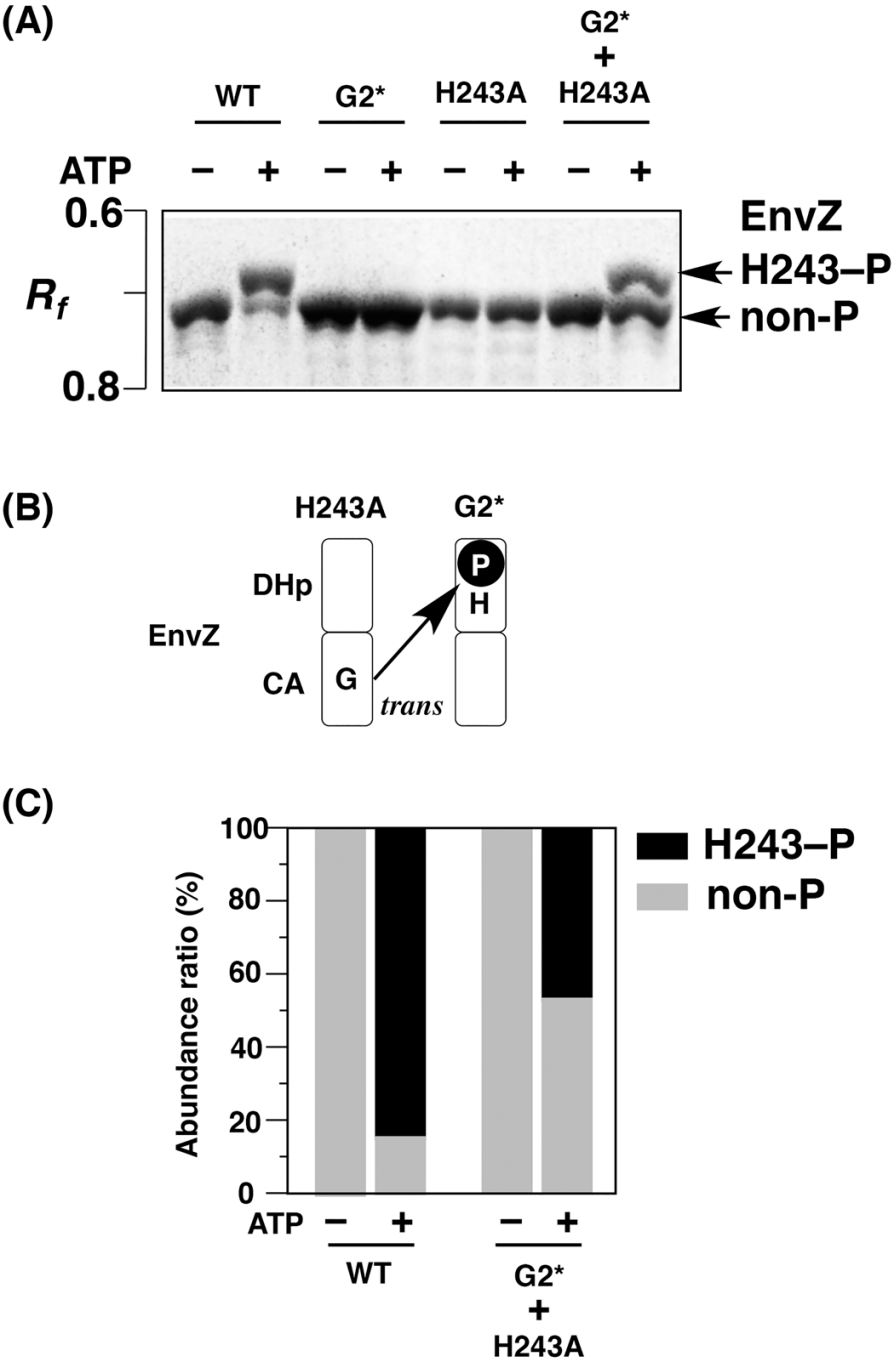
Complementation assays for autophosphorylation of mutated EnvZ proteins. (A) The autophosphorylation reactions of EnvZ wild type (WT), G2*, H243A, and an equal mixture of G2* and H243A mutants were analyzed by Phos-tag SDS-PAGE [8% (w/v) polyacrylamide and 20 μM Mn^2+^–Phos-tag]. Each lane contained 2.0 μg proteins. (B) A scheme for autophosphorylation by an intermolecular reaction from the γ-phosphate of ATP in the CA subdomain of the H243A mutant to the H243 residue in the DHp subdomain of the G2* mutant. (C) The abundance ratio of the upshifted band of H243–P in the WT lane and the G2* + H243A lane shown in (A).

We first performed an *in vitro* complementation assay of EnvZ as a typical sample that has been reported to autophosphorylate in a *trans* mode [7, 10]. We used a recombinant wild type EnvZ (WT, C-terminal 6× His-tagged protein) and two mutants. The first mutant was EnvZ G2*, in which the two glycine residues G401 and G403 in the G2 box were replaced by Ala moieties. The second was EnvZ H243A, in which the H243 autophosphorylation site was replaced with Ala. The G2 box is known to be a module of the ATP-binding region [5, 6], and the Ala-substituted mutant G2* has been used as an ATP-nonbinding variant for *in vitro* complementation assays [9, 10]. The spontaneous formation of the heterodimer of the EvnZ kinase through subunit exchange after mixing of equal parts of the two mutant homodimers of G2* and H243A mutants shows that the resulting heterodimeric mutants (G2* mutant monomer + H243A mutant monomer) can autophosphorylate in a complementary manner by using ATP as a phosphoryl donor in the *in vitro* complementation assay, as described previously [10]. The *in vitro* autophosphorylation reactions of the WT protein and its mutants were carried out in the presence of 10 mM ATP, and the reaction products were analyzed by Phos-tag SDS-PAGE (Fig 2A). The WT protein was successfully autophosphorylated, and Phos-tag SDS-PAGE permitted us to detect two migration bands: an upshifted major band corresponding to the form phosphorylated at the H243 residue (H243–P), and a minor band corresponding to the nonphosphorylated form (non-P) (see the WT lane of ATP +). In the mutants G2* and H243A, on the other hand, no upshifted band was detected, showing that the mutants did not autophosphorylate. In the autophosphorylation reaction of a sample containing equal parts of G2* and H243A mutants, we detected an upshifted band corresponding to the phosphorylated form (see the G2* + H243A lane of ATP +), showing that the facile exchange of subunits between the two mutant homodimers and the subsequent complementary autophosphorylation both occur as intermolecular reactions (Fig 2B). This result is consistent with previous reports [7, 10]. We also performed densitometric analyses to calculate the ratio of the upshifted band of H243–P to the total bands in the WT lane and the G2* + H243A lane. As shown in Fig 2C, the ratios of the phosphorylated forms of the WT protein and the mixed mutants reached values in excess of 80% and 40%, respectively, in the *in vitro* assay. Mixing of equal amounts of two mutant subunits should give rise to two homodimers and the heterodimer at a stoichiometry of 1:1:2, assuming that these subunits refold randomly. If exchange of subunits does not occur, only half the subunits in heterodimers (25% of all subunits) can possibly be phosphorylated. The results therefore indicated that the subunit-exchange reaction occurs spontaneously between the monophosphorylated homodimers through a model known as the flip-flop autokinase mechanism, as described previously [20]. Therefore, this complementation assay using the two EnvZ mutants demonstrated that Phos-tag SDS-PAGE provides a simple and reliable method for examining whether sensor kinases autophosphorylate in a *trans* manner.

### Preparation of TSKs from *E*. *coli* and their mutants, and verification of potential for phosphorylation in the mutants

**Fig 3:**
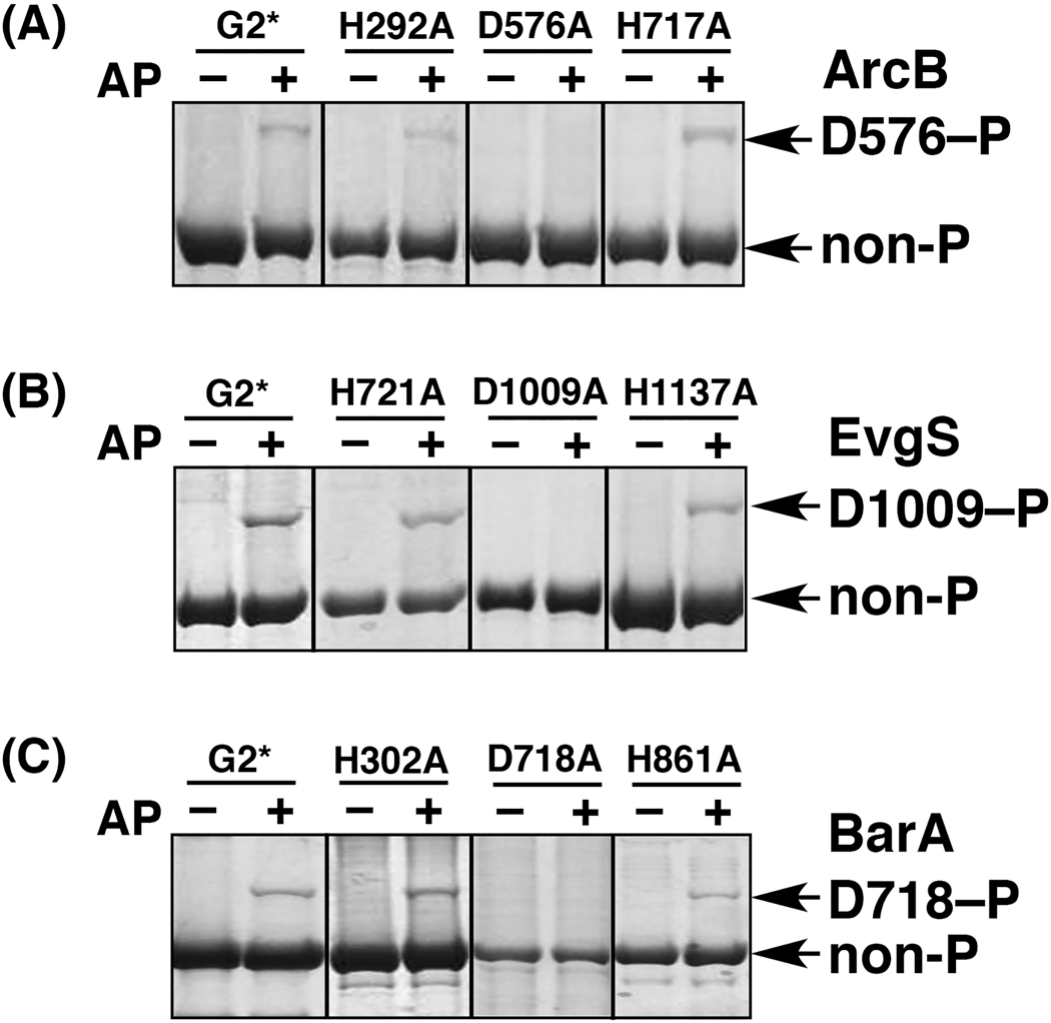
AP-dependent autophosphorylation reactions of mutated ArcB, EvgS, and BarA proteins. The G2-box-mutated proteins, ArcB G2* (A), EvgS G2* (B), or BarA G2* (C), the proteins ArcB H292A (A), EvgS H721A (B), or BarA H302A (C), which were His-mutated in the HK domain; ArcB D576A (A), EvgS D1009A (B), and BarA D718A (C), which were Asp-mutated in the receiver domain; and ArcB H717A (A), EvgS H1137A (B), and BarA H861A (C), which were His-mutated in the HPt domain, were autophosphorylated in the presence of AP, and then analyzed by Phos-tag SDS-PAGE [8% (w/v) polyacrylamide and 20 μM Mn^2+^–Phos-tag]. Each lane contained 2.0 μg proteins.

For the purpose of this study, we used three TSKs: ArcB, EvgS, and BarA. As well as the recombinant EnvZ proteins described above, we prepared their G2* mutants, which were mutated in the G2 box, and their ArcB H292A, EvgS H721A, and BarA H302A mutants, mutated at the appropriate autophosphorylation site of the DHp subdomain (see Table 2 for a summary listing the mutants that we used in this study). None of these TSKs mutated in the G2 box and the DHp subdomain were autophosphorylated in the presence of ATP (data shown below). These results were the same as those obtained for the EnvZ G2* and H243A mutants (see Fig 2A), and are reasonable. However, it worried us that these mutants might lose their potential for phosphorylation as a result of denaturation arising from the introduction of Ala substitution. To verify that these mutants retained the potential for phosphorylation, we performed AP-dependent phosphorylation assays with these mutants. Because the Asp residue in the receiver domain of TSKs can be autophosphorylated directly (rather than through the HK domain) on treatment with AP as a phosphoryl donor [19], we performed the autophosphorylation reactions of all the TSK mutants listed in Table 2 in the presence of 40 mM AP. The reaction products were then analyzed by Phos-tag SDS-PAGE (Fig 3). The mutants of G2* and the His residues in the HK and HPt domains were successfully autophosphorylated in the presence of AP; subsequent Phos-tag SDS-PAGE permitted us to detect a single upshifted band corresponding to the form phosphorylated at the Asp residue in the receiver domain of each mutant (D576–P of ArcB in A, D1009–P of EvgS in B, and D718–P of BarA in C; see the appropriate lanes of AP +) in the same manner as in our previous report, in which we used the same mutants or the WT protein [19]. In a consistent manner, no upshifted bands were detected in the mutants of the Asp residue in the receiver domain. Because the autophosphorylation reaction of the Asp residue in the presence of AP occurs specifically through enzymatic activities [21], these results confirmed that the mutant TSKs that we used had the potential to undergo phosphorylation and were therefore confirmed to be suitable for use in complementation assays to determine whether the corresponding TSKs autophosphorylate in a *cis* manner. Note, however, that these mutants might not be completely folded, even though they retain enzymatic activities for phosphorylation with AP as a phosphoryl donor.

**Table 2:** List of the mutant TSKs used in this study.

TSK	Substituted domain or region			
	G2 box	DHp	Receiver	HPt
ArcB	G2* (G470A, G472A)	H292A	D576A	H717A
EvgS	G2* (G879A, G881A)	H721A	D1009A	H1137A
BarA	G2* (G481A, G483A)	H302A	D718A	H861A

### Autophosphorylation and multistep phosphotransfer modes of ArcB

**Fig 4:**
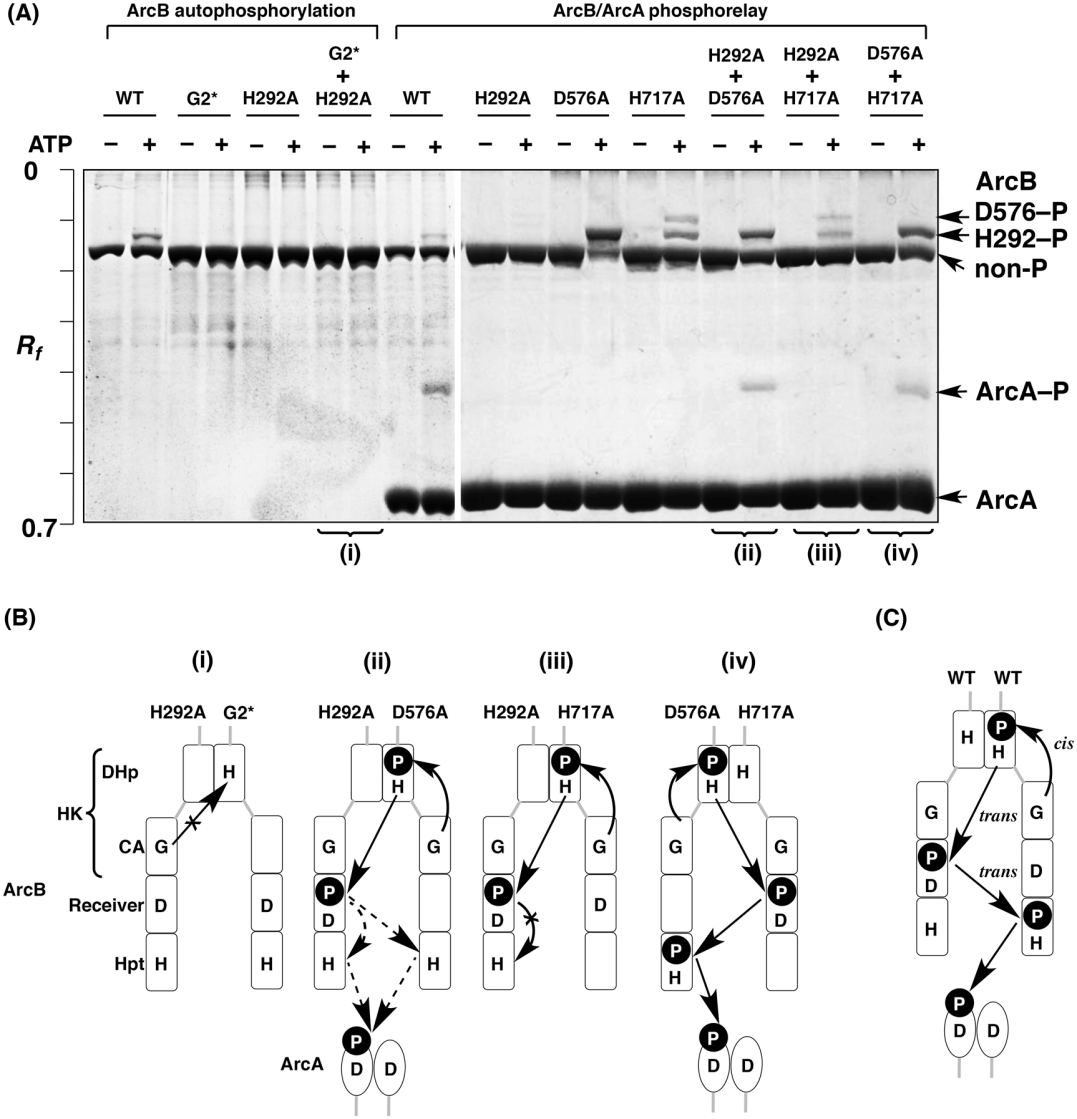
Complementation assays for mutated ArcB proteins. (A) The reactions of ArcB autophosphorylation and ArcB/ArcC phosphorelay were analyzed by Phos-tag SDS-PAGE [8% (w/v) polyacrylamide and 20 μM Mn^2+^–Phos-tag]. Each lane contained 2.0 μg of ArcB or ArcC. (B and C) Schemes of modes of autophosphorylation and phosphoryl-transfer reactions, based on the Phos-tag SDS-PAGE gel images shown in A.

Next, we performed a complementation assay between the ArcB G2* and H292A mutants to confirm the intramolecular nature of the autophosphorylation of ArcB, as reported previously [9]. It has been demonstrated that heterodimers of ArcB are also formed by mixing the G2* and H292A mutants under the conditions for the *in vitro* complementation assay. *In vitro* autophosphorylation reactions were carried out in the presence of 10 mM ATP, and the reaction products were analyzed by Phos-tag SDS-PAGE (see the lanes for ArcB autophosphorylation in Fig 4A). The WT protein was successfully autophosphorylated, and Phos-tag SDS-PAGE permitted us to detect an upshifted band corresponding to the autophosphorylated ArcB form H292–P containing a phosphorylated H292 residue (indicated by the arrow on the right-hand side of Fig 4A), as described previously [19]. On the other hand, no upshifted band was detected in the G2* or H292A mutants, showing that these mutants do not autophosphorylate, as described above. In the autophosphorylation reaction using a mixed sample of G2* and H292A mutants in equal proportions, no upshifted band was detected, once more showing that complementary autophosphorylation between the two mutants did not occur [Fig 4B(i)]. The complementation assay therefore demonstrated that ArcB autophosphorylates in a *cis* mode (Fig 4C), as reported previously [9].

To determine the mode of the subsequent multistep phosphorelay, we performed three additional complementation assays between the ArcB H292A and D576A mutants, the H292A and H717A mutants, and the D576A and H717A mutants, respectively, in the presence of ArcA (see lanes for the ArcB/ArcA phosphorelay in Fig 4A). We observed a single upshifted band corresponding to the phosphorylated ArcA (ArcA–P) in the phosphorelay reaction with the ArcB WT, showing that a phosphoryl-transfer reaction from the ArcB WT to ArcA had occurred. Furthermore, as reported previously [19] for the phosphorelay reaction with the H292A mutant, no upshifted band of ArcB was observed, whereas a strong single upshifted band corresponding to the phosphorylated form H292–P was detected in the phosphorelay reaction with the D576A mutant, and two upshifted bands corresponding to the phosphorylated forms H292–P and D576–P were detected in the phosphorelay reaction with the H717A mutant. In the phosphorelay reaction using an equal mixture of H292A and D576A mutants in the presence of ArcA, an upshifted band corresponding to the autophosphorylated ArcB of H292–P was observed, and the phosphoryl group was transferred to ArcA. This result demonstrated that exchange of subunits between the two dimeric ArcB mutants (H292A and D576A mutants) readily occurs in the same manner as for EnvZ, as described above, and that the complementary phosphotransfer reaction from the H292 residue to the D576 residue occurs intermolecularly (the *trans* mode) [Fig 4B(ii)], indicating that the dimeric ArcB protein, like the EnvZ protein, is thermodynamically stable but kinetically labile. In the phosphorelay reaction using an equal mixture of H292A and H717A mutants, on the other hand, although two upshifted bands corresponding to the phosphorylated ArcB of H292–P and D576–P were observed, no upshifted band of ArcA was detectable. Furthermore, in the phosphorelay reaction using an equal mixture of D576A and H717A mutants, we detected a single upshifted band of ArcA. These results indicate that the phosphoryl-transfer reaction from the D576 residue to the H717 residue proceeds through a *trans* mode [Fig 4B(iii) and 4B(iv)]. We therefore concluded that the primary ArcB autophosphorylation reaction occurs as an intramolecular reaction (*cis* mode) and the subsequent His–Asp–His phosphorelay reactions occur as intermolecular reactions (*trans* modes) (see Fig 4C); in other words, the three-step phosphorelay of ArcB proceeds in a *cis*-*trans*-*trans* mode.

### Autophosphorylation and multistep phosphotransfer modes of EvgS

**Fig 5:**
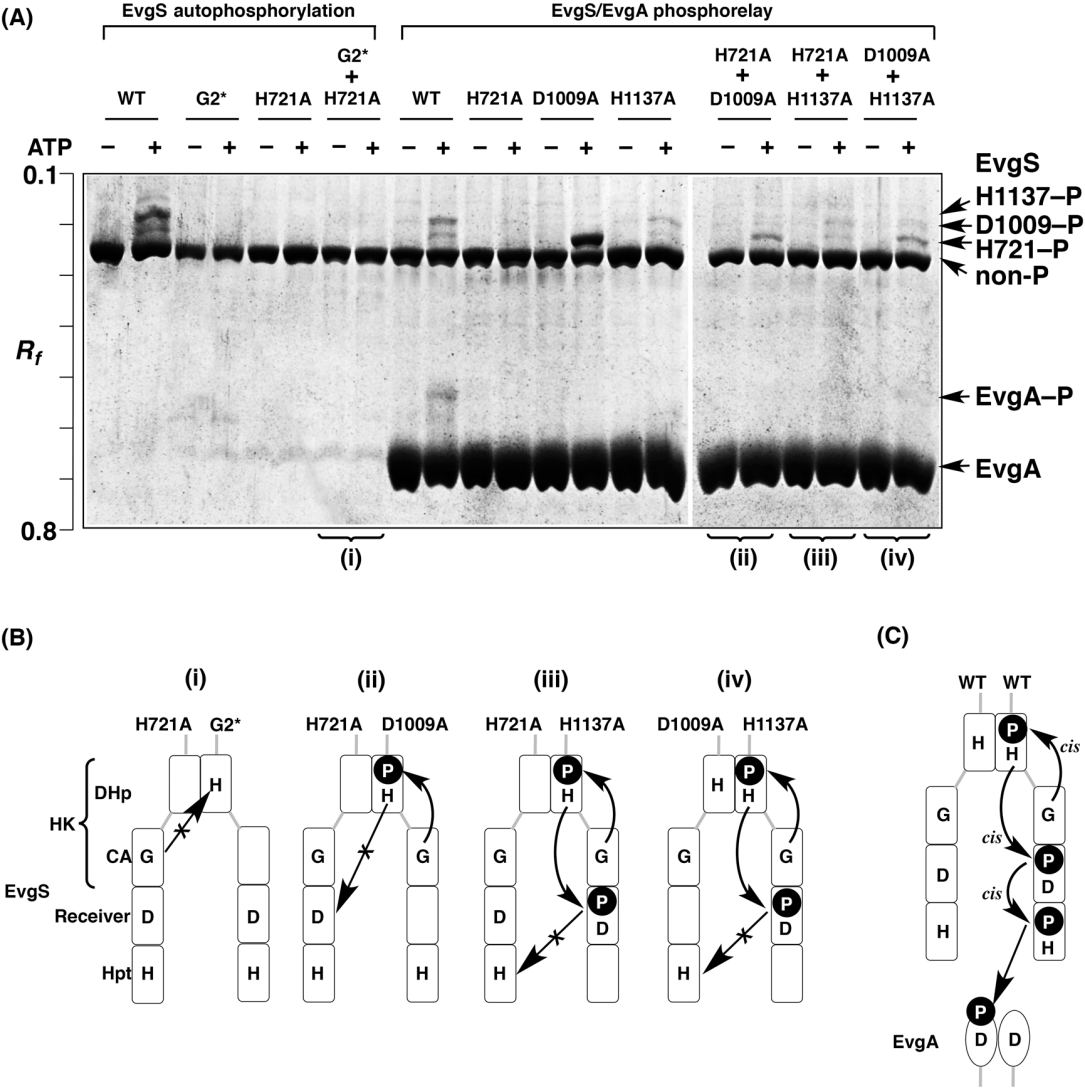
Complementation assays for mutated EvgS proteins. (A) The reactions of EvgS autophosphorylation and EvgS/EvgA phosphorelay were analyzed by Phos-tag SDS-PAGE [8% (w/v) polyacrylamide and 20 μM Mn^2+^–Phos-tag]. Each lane contained 2.0 μg of EvgS or EvgA. (B and C) Schemes of modes of autophosphorylation and phosphoryl-transfer reactions, based on the Phos-tag SDS-PAGE gel images shown in A.

We performed a similar complementation assay between the EvgS G2* and H721A mutants to determine whether EvgS autophosphorylate in a *cis* or a *trans* mode. *In vitro* autophosphorylation reactions were carried out in the presence of 30 mM ATP, and the reaction products were analyzed by Phos-tag SDS-PAGE (see the lanes for EvgS autophosphorylation in Fig 5A). The WT protein was successfully autophosphorylated, and Phos-tag SDS-PAGE permitted us to detect three upshifted bands corresponding to the phosphorylated forms of EvgS. As described previously [19], we assigned the low-mobility, medium-mobility, and high-mobility bands to the phosphorylated forms H1137–P (with a phosphorylated H1137 residue), D1009–P (with a phosphorylated D1009 residue), and H721–P (with a phosphorylated H721 residue), respectively (indicated by the arrows on the right-hand side of Fig 5A). In addition, we have demonstrated that it is unlikely that a phosphorylated form having two or more phosphoryl groups is produced in each EvgS subunit [19]. On the other hand, the G2* and H721A mutants did not autophosphorylate as described above. Furthermore, in the autophosphorylation reaction with an equal mixture of G2* and H721A mutants, no upshifted band was observed, showing that no complementary autophosphorylation between the two mutants had occurred [see Fig 5B(i)]. The complementation assay therefore showed that EvgS autophosphorylates in a *cis* mode, in the same way as ArcB (Fig 5C).

To determine the mode of the subsequent multistep His–Asp–His phosphorelay, we performed another three complementation assays between the EvgS H721A and D1009A mutants, the H721A and H1137A mutants, and the D1009A and H1137A mutants, respectively, in the presence of EvgA (see the lanes for the EvgS/EvgA phosphorelay in Fig 5A). We observed only a single upshifted band corresponding to the phosphorylated EvgA (EvgA–P) in the phosphorelay reaction with the EvgS WT, showing that a phosphoryl-transfer reaction from the EvgS WT to EvgA occurs. The low-mobility band corresponding to the phosphorylated form H1137–P, observed to a slight extent in the absence of EvgA (that is, in the autophosphorylation reaction of EvgS), was not detectable in the presence of EvgA because of the rapid phosphoryl-transfer reaction from the H1137 residue to EvgA, in the same manner as described in our previous report [19]. Furthermore, as reported previously [19], for the phosphorelay reaction with the H721A mutant, no upshifted band of EvgS was observed, whereas a strong single upshifted band corresponding to the phosphorylated form H721–P was detected in the phosphorelay reaction with the D1009A mutant, and two upshifted bands corresponding to the phosphorylated forms H721–P and D1009–P, similar to those with the EvgS WT, were detected in the phosphorelay reaction with the H1137A mutant. In the phosphorelay reaction using an equal mixture of H721A and D1009A mutants in the presence of EvgA, only an upshifted band corresponding to the autophosphorylated EvgS of H721–P was observed, and the phosphoryl group was not transferred to EvgA. This result demonstrated that the phosphotransfer reaction from the H721 residue to the D1009 residue occurs as an intramolecular reaction (*cis* mode) [Fig 5B(ii)]. Similarly, we can explain the two complementation assays using mixed samples of H721A/H1137A mutants and D1009A/H1137A mutants in the presence of EvgA. In other words, both results indicate that an intramolecular phosphoryl-transfer reaction from the D1009 residue to the H1137 residue occurs [Fig 5B(iii) and 5B(iv)]. We therefore concluded that the three-step phosphorelay of EvgS proceeds by a *cis*-*cis*-*cis* mode (see Fig 5C).

### Autophosphorylation and multistep phosphotransfer modes of BarA

**Fig 6:**
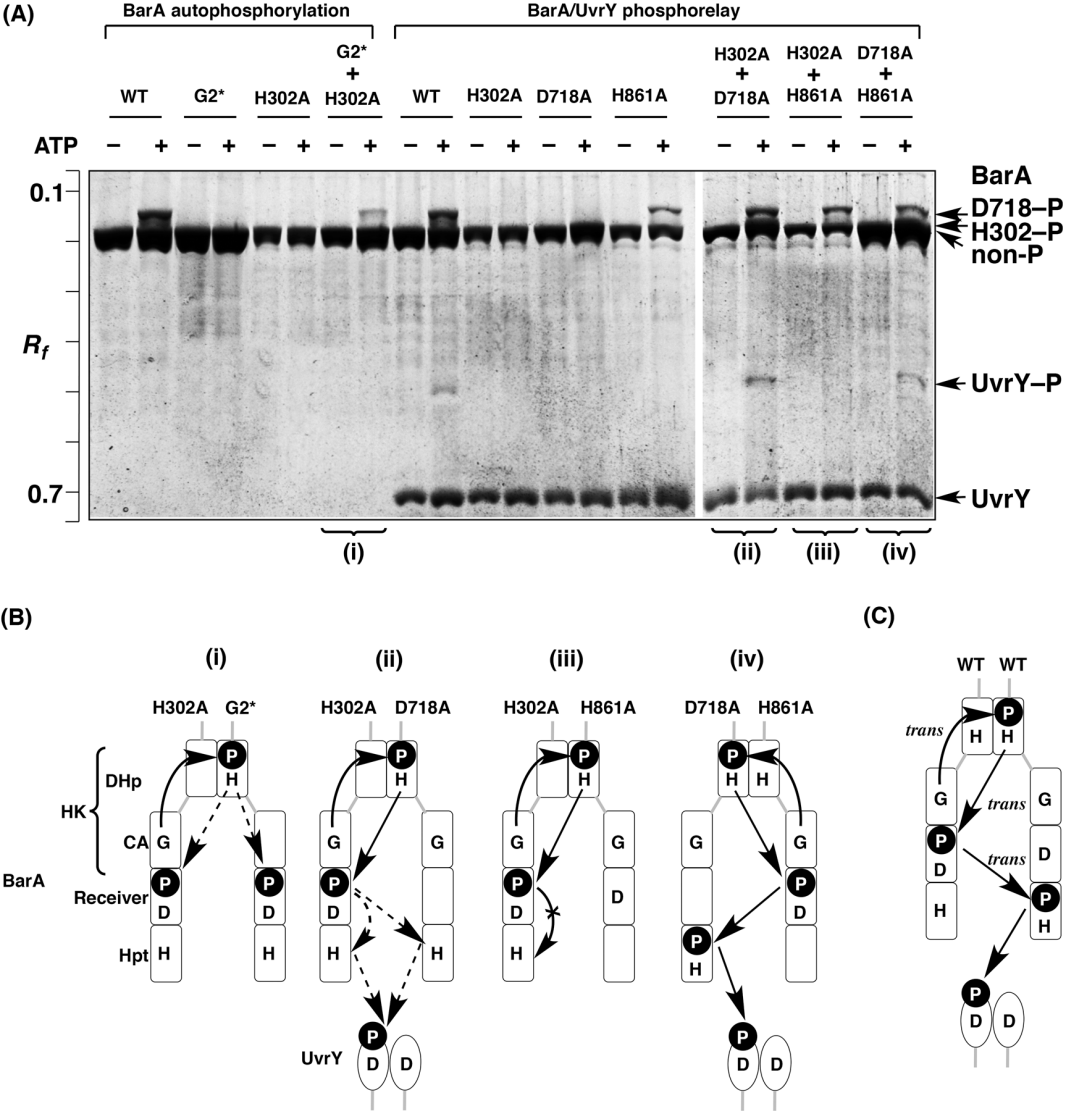
Complementation assays for mutated BarA proteins. (A) The reactions of BarA autophosphorylation and BarA/UvrY phosphorelay were analyzed by Phos-tag SDS-PAGE [8% (w/v) polyacrylamide and 20 μM Mn^2+^–Phos-tag]. Each lane contained 2.0 μg of BarA or UvrY. (B and C) Schemes of modes of autophosphorylation and phosphoryl-transfer reactions, based on the Phos-tag SDS-PAGE gel images shown in A.

Finally, we performed a complementation assay between the BarA G2* and H302A mutants to determine whether BarA autophosphorylates in a *cis* or *trans* manner. *In vitro* autophosphorylation reactions were carried out in the presence of 10 mM ATP, and the reaction products were analyzed by Phos-tag SDS-PAGE (see the lanes for BarA autophosphorylation in Fig 6A). The WT protein was successfully autophosphorylated, and Phos-tag SDS-PAGE permitted us to detect two upshifted bands corresponding to the phosphorylated forms H302–P and D718–P. As previously reported [19], there was only a small difference between the degrees of migration of the phosphorylated BarA of H302–P and that of the nonphosphorylated form (non-P). The G2* and H302A mutants did not autophosphorylate as described above. In the autophosphorylation reaction of an equal mixture of G2* and H302A mutants, on the other hand, we observed a clear upshifted band corresponding to the phosphorylated form D718–P, showing that a complementary autophosphorylation reaction occurs as an intermolecular reaction and the following phosphoryl-transfer reaction from the H302 residue to the D718 residue occurs as an intra- or intermolecular reaction [Fig 6B(i)]. The complementation assay therefore demonstrated that BarA autophosphorylates in a *trans* manner (Fig 6C).

To confirm and identify the mode of the subsequent multistep phosphorelay, we performed three more complementation assays between the BarA H302A and D718A mutants, the H302A and H861A mutants, and the D718A and H861A mutants, respectively, in the presence of UvrY (see the lanes for the BarA/UvrY phosphorelay in Fig 6A). We observed a single upshifted band corresponding to the phosphorylated UvrY (UvrY–P) in the phosphorelay reaction with the BarA WT, showing that a phosphoryl-transfer reaction occurs from the BarA WT to UvrY. In the phosphorelay reaction using an equal mixture of H302A and D718A mutants in the presence of UvrY, a clear upshifted band corresponding to the phosphorylated BarA of D718–P was observed, and the phosphoryl group was transferred successfully to UvrY. This result confirmed that the complementary phosphotransfer reaction from the H302 residue to the D718 residue proceeds as an intermolecular reaction (*trans* mode) [Fig 6B(ii)]. However, in the phosphorelay reaction using an equal mixture of H302A and H861A mutants, although a clear upshifted band corresponding to the phosphorylated BarA of D718–P was observed, no upshifted band for UvrY was visible. Furthermore, in the phosphorelay reaction using an equal mixture of D718A and H861A mutants, we detected a single upshifted band of UvrY. These results indicate that the phosphoryl-transfer reaction from the D718 residue to the H861 residue occurs in a *trans* mode [Fig 6B(iii) and 6B(iv)]. We therefore concluded that the three-step phosphorelay of BarA proceeds in a *trans*-*trans*-*trans* mode (see Fig 6C).

## Discussion

We have demonstrated a simple strategy for determining whether autophosphorylation and phosphoryl-transfer reactions in TSKs occur in a *cis* mode or in a *trans* mode by using an *in vitro* complementation assay followed by Phos-tag SDS-PAGE analysis. The phosphate-affinity electrophoresis permitted us to identify the phosphorylation mode of three types of TSKs (ArcB, EvgS, and BarA) derived from *E*. *coli*. The autophosphorylation reactions of ArcB and EvgS occur as intramolecular reactions. Our result for ArcB is consistent with a previous report [9]. In the case of EvgS, the intramolecular autophosphorylation was an unexpected result, because the intramolecular reaction departs from the usual model of intermolecular autophosphorylation of homodimeric sensor kinases [2]. Actually, the autophosphorylation mode of BvgS from *B*. *pertussis*, which bears a close resemblance to EvgS [22, 23], had been previously described as proceeding in a *trans* mode [13, 19]. In addition to the new finding relating to the intramolecular autophosphorylation of EvgS, we presume that the *cis* autophosphorylation mode might occur with other sensor kinases.

It is interesting that the individual schemes of the phosphorelay reactions of these TSKs are completely different. In the case of EvgS, we concluded that all steps of the phosphorelay from the primary autophosphorylation reaction in the HK domain to the terminal phosphotransfer to the HPt domain proceed through *cis* modes (see Fig 5C). With BarA, in contrast, all proceed through *trans* modes (see Fig 6C). Although ArcB autophosphorylates in a *cis* mode in the same way as EvgS, the subsequent His–Asp–His phosphorelay reactions occur as intermolecular reactions (see Fig 4C). The *trans* mode observed in ArcB and BarA is reasonable, given that these kinases function as homodimers, as suggested by the previous report [2], whereas the *cis*-*cis*-*cis* mode throughout the phosphorelay of EvgS disclosed by this study is of particular interest to us. In our previous report [19], we indicated that the EvgS D1009A mutant forms a dimer under the same experimental conditions as those of the present study and that the homodimeric mutant autophosphorylates by a flip-flop autokinase mechanism, in which only one subunit can be phosphorylated in an autophosphorylation reaction [20]. Consistently, we also observed in this study that the D1009A–P phosphorylated form (that is, the high-mobility form of the D1009A mutant with a phosphorylated H721 residue) was produced in excess in the phosphorelay reaction (see D1009A in Fig 5A, lane of ATP +), indicating that the subunit-exchange reaction occurs as an intermolecular reaction in the homodimeric EvgS, as well as the EnvZ WT protein (see Fig 2C). In considering the functional relevance of dimerization, the *cis*-*cis*-*cis* mode of EvgS might be a key phenomenon. Furthermore, it has been reported that some recombinant TSKs, including the dimeric EvgS, have a clear tendency to undergo self-association and clustering in *E*. *coli* cell membranes [24]. The functional role of behaviors of TSKs such as dimerization or clustering under natural conditions awaits further study.

Meanwhile, our conclusion regarding the *cis* mode in this study should be treated with caution. We have only provided evidence that *trans* phosphorylation can occur, but have not provided direct evidence for the occurrence of *cis* phosphorylation. For instance, when we used the two EvgS mutants of G2* and H721A in the *in vitro* complementation assay, there was no autophosphorylation (see Fig 5A). From this negative result, we concluded that *cis* phosphorylation must have occurred in EvgS, without consideration of other reasons how the negative results might have been obtained. Note that negative results might also have occurred for other reasons. For example, if EvgS forms homodimers with higher affinity, there would effectively be no formation of a heterodimer (and no phosphotransfer); alternatively, mutation of G2* or H721A in EvgS might cause misfolding of the HK domain (while leaving the receiver domain intact), which would also result in a negative result with respect to *trans* phosphorylation. These are innate limitations of molecular biological experiments that involve site-directed mutagenesis.

Recently, a combined study involving molecular and genetic approaches, coupled with mathematical and statistical modeling, demonstrated that the phosphorelay of ArcB *in vivo* proceeds by a bimolecular mechanism; that is, the phosphorylation mode was not identified as an exclusive *cis* or *trans* mode [25]. In contrast, the analysis of *in vitro* reaction products by Phos-tag SDS-PAGE permitted us to identify whether each of the three phosphoryl-transfer reaction steps occurs in an exclusive *cis* or *trans* mode. Phos-tag SDS-PAGE therefore provides a simple method for identifying the unique and specific phosphotransfer mode for a given kinase, without taking complicated intracellular elements into consideration.
